# Eye Movements, Visual Search and Scene Memory, in an Immersive Virtual Environment

**DOI:** 10.1371/journal.pone.0094362

**Published:** 2014-04-23

**Authors:** Dmitry Kit, Leor Katz, Brian Sullivan, Kat Snyder, Dana Ballard, Mary Hayhoe

**Affiliations:** 1 Computer Science Department, University of Bath, Bath, United Kingdom; 2 Institute for Neuroscience, Center for Perceptual Systems, University of Texas, Austin, Texas, United States of America; 3 Smith Kettlewell Eye Research Institute, San Francisco, California, United States of America; 4 Psychology Department, Center for Perceptual Systems, University of Texas, Austin, Texas, United States of America; 5 Computer Science Department, Center for Perceptual Systems, University of Texas, Austin, Texas, United States of America; Barrow Neurological Institute, United States of America

## Abstract

Visual memory has been demonstrated to play a role in both visual search and attentional prioritization in natural scenes. However, it has been studied predominantly in experimental paradigms using multiple two-dimensional images. Natural experience, however, entails prolonged immersion in a limited number of three-dimensional environments. The goal of the present experiment was to recreate circumstances comparable to natural visual experience in order to evaluate the role of scene memory in guiding eye movements in a natural environment. Subjects performed a continuous visual-search task within an immersive virtual-reality environment over three days. We found that, similar to two-dimensional contexts, viewers rapidly learn the location of objects in the environment over time, and use spatial memory to guide search. Incidental fixations did not provide obvious benefit to subsequent search, suggesting that semantic contextual cues may often be just as efficient, or that many incidentally fixated items are not held in memory in the absence of a specific task. On the third day of the experience in the environment, previous search items changed in color. These items were fixated upon with increased probability relative to control objects, suggesting that memory-guided prioritization (or Surprise) may be a robust mechanisms for attracting gaze to novel features of natural environments, in addition to task factors and simple spatial saliency.

## Introduction

How are visual scenes represented in memory? Evidence is accumulating that such representations are multi-faceted. There is consensus that the ‘gist’ of a scene is rapidly perceived and retained in memory, along with limited short term visual memory of a few items, and other high level semantic information [1]–[3]. The change blindness phenomenon was initially interpreted to mean that little else was retained beyond this (see review in [4]). Some of the evidence was taken to indicate that coherent object representations decayed rapidly following withdrawal of attention from an object [5], [6]. Wolfe also argued for the decay of object representations following withdrawal of attention [7]. More recent work, however, has suggested that there is a much more extensive accumulation of information in memory even after relatively brief exposures [8]–[12]. For example, scene context appears to facilitate subsequent visual search for targets even after a single prior exposure [13]–[15]. Thus it appears that the change blindness phenomenon leads to an underestimate of the extent of visual scene representations [8], [10], [13].

The nature of visual scene representations will vary with the extent of prior visual experience. Many experiments in scene perception, including many change blindness experiments, entail a sequence of limited exposures to a relatively large number of images of natural scenes. Normal visual experience, however, is quite different, and typically involves long periods of immersion in a relatively small number of visual environments, such as one's home, workplace, etc. Even though attention and working memory may limit the information acquired in any given fixation, after many thousands of fixations within the same scene (about 10,000 per hour), there is ample opportunity to accumulate highly detailed statistical representations. It is well established that the human perceptual system is highly sensitive to these statistics and that these learnt priors about scenes play an important role in perception [16], [17]. Thus memory for the scenes that constitute a large fraction of ordinary visual experience may function in a somewhat different manner from memory for generic scenes typically used in laboratory experiments on scene memory. Another difference between the laboratory and ordinary visual experience is that the latter often incurs physical effort (e.g. walking towards an object of interest), a process that may affect scene memory. Observing behavior in immersive environments is therefore integral to furthering our understanding of the role of memory in natural vision [18], [19].

The goal of the present study was to understand the role that scene memory might play in the allocation of attention and eye movements in natural, ordinary vision, and appreciate how it may differ from laboratory settings. The traditional solution to understanding the control of attention and gaze has been to assume that some ongoing ‘pre-attentive’ analysis of the visual image takes place, and that the products of this analysis attract the observer's attention to important or salient aspects of the image for further processing [7], [20], [21]. There has been extensive investigation of the role of stimulus salience in attracting attention (e.g., [22]–[25]). However, evidence for the role of salient stimuli in attracting attention is mixed (see reviews [26], [27]). Such mechanisms are inherently brittle as they rely on the properties of the stimulus with respect to the immediate context to attract attention. Even if stimulus salience, defined in this way, plays some role in attracting gaze in laboratory experiments, it is uncertain whether these mechanisms will be very effective in natural environments, since the experimental contexts examined may not reflect either the sensory milieu of the natural world or the requirements of visually guided behavior. In natural behavior, many kinds of information need to be attended, and important information may not be especially salient (for example, irregularities in the sidewalk). Conversely, salience may not be especially important and may not attract attention [28]–[30]. At the same time there is a clear need for observers to detect new or unexpected aspects of familiar scenes. There must be some mechanism for attracting attention to aspects of the scene that are not on the current task agenda and require a change of task priorities, such as avoiding an unexpected obstacle. It is in this context that scene memory may play an important role. The problem in natural vision is that a stimulus that is salient in one context (such as peripheral motion with a stationary observer) may not be salient in another context (such as when the observer is moving and generating complex motion patterns on the retina). However, if the scene is efficiently coded in long-term memory, different mechanisms might be available for coding new information. Subjects may compare the current image with the stored representation, and a mis-match, or “residual” signal may serve as a basis for attracting attention to changed regions of scenes [31]. This may allow subjects to be particularly sensitive when there are deviations from familiar scenes, and thus attention may be drawn to regions that do not match the stored representation.

This idea is similar in conception to Itti and Baldi's [32], [33] model of “Surprise”. Horstmann [34], [35] and Becker et al. [36] also showed that distractors that have not been previously presented (i.e., are surprising) attract attention in a visual search task. Itti and Baldi conjecture that the visual system learns the statistics of images by estimating the distribution of parameters of probability distributions that can explain recent image feature data. Itti and Baldi's model works on very short time scales (100's of msec). Thus it is unlikely to reflect the long-term memory factors involved in natural behavior. An alternative mechanism may be one that relies on learnt statistics of scenes, and is sensitive to scene regions that differ significantly from these statistics. In standard saliency models, salient stimuli are statistical outliers in space. Surprising stimuli can be thought of as statistical outliers with respect to learnt, expected, distributions stored in memory (cf. [37], [38]). Such a mechanism might serve as a more robust mechanism of attentional capture than purely spatial saliency.

Evidence that memory representations facilitate detection of novel objects in scenes was found by Brockmole and Henderson [39], [40]. When subjects were given 15 seconds pre-exposures to images of natural scenes, new objects were able to attract gaze in a subsequent brief exposure, even when the object was presented during a saccade, and there was no retinal transient associated with its appearance. The authors suggest that the pre-exposure allowed subjects to construct a long-term memory representation of the scene, as a basis for discriminating the new object. Subsequent experiments revealed that inconsistent objects had greater attentional prioritization than consistent object [41] and that color changes were also prioritized following a preview [42]. Thus when the scene is familiar, changes may be more readily detectable. Brockmole and colleagues refer to this as “memory-guided prioritization”.

Although these experiments used images of natural scenes, the nature of the experience still differs from natural vision, as described above, with respect to the number of scenes, and the temporal sequence. Only a few studies have examined scene memory or gaze allocation in realistic, immersive and complex natural environments. Droll et al. [43] and Tatler et al. [44] demonstrated the role of the task in gaze allocation and memory, although there is also evidence for memory that is not obviously related to the instructed task. Mack et al. [45] showed that previously acquired associative knowledge influences gaze allocation and facilitates search in real world scenes. In a previous study in an immersive virtual environment, Karacan et al. [46] attempted to demonstrate the role of prior experience, where observers walked around a virtual environment for several minutes. Subjects with this experience in the environment looked more at changed objects than those without experience. All of these studies, however, used relatively short exposures.

The current study was designed to examine the effect of more prolonged exposure to such an environment. Rather than a few minutes of experience we gave subjects experience in the environment over three sessions on separate days, adding up to about one hour's total experience. In addition to studying the role of memory in guiding eye movements, we tested whether the prolonged experience facilitated the detection of changed objects and allocation of gaze to them. Although the experiment in [46] was consistent with such a finding, they were not able to demonstrate an increase in the probability of fixation on the changed objects, but only an increase in total fixation duration on the objects. It was not clear if the longer fixation duration was a result of attentional capture (or gaze prioritization in the terminology of [39], [40]) or simply a consequence of longer fixations once gaze was actually on the object. In the present experiment, therefore, we asked whether experience in a scene might form the basis of a mechanism that attracts gaze to regions that differ from the memory representation. Such a mechanism might be more robust in attracting gaze to regions that are not currently task relevant than stimulus saliency.

In addition to the potential role of scene memory in detecting changes, another important function is visual search. Evidence is accumulating that pre-exposure to images of natural scenes facilitates subsequent search [8], [12]–[14], [47], [48]. However, it is not known how much search benefits from extensive immersive experience in a naturalistic scene. In 2D natural images, search times for ordinary objects are typically 1–2 sec, and involved 3–5 fixations [49], [50]. There is a small advantage to repeated searches for different objects within the same scene, at least when the target is specified by a verbal label, and a bigger advantage for repeated searches of the same object. In an immersive environment, the search item will typically be at a remote location in the space and not visible to the subject at the initiation of search, so subjects need to learn the location of the item in the larger space, and use memory for layout in order to bring the object within view. To test both these functions of visual memory in immersive environments, namely search and change detection, we designed a three-room virtual apartment, and asked subjects to search for a sequence of targets over three sessions on subsequent days. A small number of these objects were specified as search targets on repeated occasions, and this allowed us to explore the effect of repeated searches, and the effect of scene memory on this search process. In the final session the color of some items was changed, and we asked whether the probability of fixation on these items increased following the change.

## Methods

### Experimental Environment

**Figure 1:**
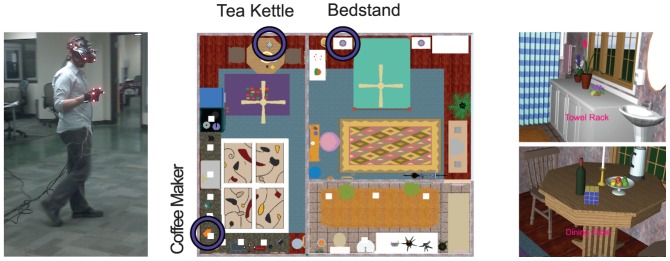
Subjects performed a visual search task within a virtual-reality three-room apartment. A. Subjects wore a V8 head mounted display (HMD) equipped with an ASL eye tracker, while the head and hand were tracked using the PhaseSpace motion tracking system. The subject touched virtual objects in the apartment using the gloved hand when they were located. B. Overhead view of the virtual apartment showing the three rooms: bathroom, bedroom, and kitchen. The 3 objects specified for repeated search are indicated by purple circles. C. Two example views, recorded while a human subject was exploring the apartment wearing the HMD. Targets were specified by words at the bottom of the screen (e.g., “Coffee Maker”) and the subject had to locate and touch that object (e.g., the orange coffee maker). Gaze position, head orientation and location were recorded for later analysis.

Participants were asked to explore a virtual reality (VR) apartment, created with IMSI FloorPlan 3D V11, consisting of three rooms: bedroom, bathroom and kitchen. A top view of the environment is shown in Figure 1A. The subjects wore a Virtual Research V8 head mounted display (HMD). This HMD has two 640×480 pixel resolution screens and a 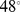
x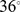 field of view. Fixed to it were 6 LED markers, which were tracked by a PhaseSpace motion tracking system at 480 Hz and used to monitor head position in the environment and to update the view of the environment at the 60 Hz refresh rate of the HMD. The HMD display was updated based on the orientation and position of the rigid body defined by these markers in a 4.6 m by 5.5 m space. This information was recorded for later analysis. In addition, each subject wore a glove with an LED marker on the index finger, which was also tracked by the PhaseSpace System. A view of the subject walking in the virtual environment is shown in Figure 1B. An Applied Science Laboratory (ASL) eye tracker recorded the position of the left eye at a sampling rate of 60 Hz and an accuracy of approximately one degree. Before the experiment the eye tracker was calibrated using a nine point (3×3) grid. The quality of the track was checked and recorded at the middle and at the end of every session. If a drift was detected at the middle of the experiment the eye tracker was re-calibrated to maximize precision. Video records of the eye and scene camera were combined in a custom QuickTime digital format, which also allows the data from the head, eye-position, and finger position, and the simulation (e.g., position of objects in the world) to be saved as synchronized metadata on each video frame. To find the 3D coordinates of a fixation, as well as the identity of the fixated objects, the recorded metadata was used to reconstruct the visual experience of a subject offline. A rectangular window of 60×60 pixels was centered about the eye position location and any objects that projected onto this window were recorded as potential targets of a fixation. The object with the largest number of pixels within this window was chosen as the fixated target.

#### Ethics Statement

Six members participated in the experiment. The experiment was approved by the University of Texas Institutional Review Board (IRB: 2006-06-0085) and all participants gave written informed consent. All participants had 20/20 vision, either corrected or uncorrected, and were naive with respect to the hypotheses under investigation.

#### Procedure

The primary task was to search for objects in the apartment and to touch them when they had located them. Numerous objects were placed in this environment ranging from furniture (e.g., a desk and a bed) to appliances (e.g., blender and refrigerator). The search target was identified by words presented inside the HMD and subjects were required to locate and touch the object. After contact was recorded, a new target was displayed. Three sessions took place over three consecutive days. The duration of each of the first two sessions (excluding set-up and calibration) was approximately 20 minutes. The third session was about 8 minutes long and was used to test subject's ability to detect changes. The subjects were told that: 1. They would spend three sessions in the environment over three consecutive days. 2. The goal of the experiment was to test their ability to remember visual features of the environment. 3.There would be a test after the third session. The instructions purposefully neglected to specify what visual aspects of the environment the participants would be tested on.

The primary purpose of the search task was to give subjects experience that engaged them in exploration of the environment. During the first session the subject searched for 75 objects, 31 of which were used as targets on more than one occasion, and 44 of which were targets only once. During the second session the participants searched for 100 objects, 55 of which were targets only once. In order to investigate the effect of experience on search time, three objects were selected for repeated Search Episodes over the two sessions: coffee maker, bed stand, and tea-kettle. We refer to the 1st Search Episode as the 1st time all three objects were searched for, the 2nd Search Episode as the 2nd time all three objects were searched for, and so on. Within every Search Episode the order in which the three objects were searched for could take 1 of 6 forms, and these were arbitrarily assigned to the Search Episodes of the subjects, with little variability. Following experiments would benefit from increasing this variability by randomizing the order of search objects within and between Search Episodes and subjects in order to assert that any observed effect is not a result of the particular order of particular objects. Even though we failed to explicitly randomize the object order, this concern is mitigated by the slight differences in sequence order between and within subjects in our design.

Subjects were able to complete the second session in about the same time period as on the first day, despite the greater number of target objects. In the third session subjects continued searching for objects, including the three repeated objects. On the 25th trial the color of the tea kettle on the dining room table was changed. On the 27th trial the coffee maker changed in color and finally the bed stand changed on trial 34. These changes took place when the subject was in a different room and the object outside the field of view. The third session was terminated on trial 60, after which subjects were given a questionnaire. They were asked to sketch the environment including as many objects as they could remember. They were also asked if they noticed any objects changing and if so, what those changes were.

### Data analysis

During the experiment a data file was generated that contained positions of the subject's head and eye as well as the positions of all of the objects in the environment. By using the head orientation and position of the subject along with the positions of all the objects in the environment, the analysis tool created a complete reconstruction of the experimental environment. Subjects' eye position data were analyzed using an automated system developed in-house. The eye signal was preprocessed using a median filter followed by a moving average over three frames to smooth the signal. Eye-movement data was then segmented into fixations and saccades. Fixations were defined by sequences of at least 150 ms in length where the eye velocity was below an adaptive threshold of 40 deg/s plus half the moving average velocity calculated over 7 frames. Low velocity movements that are a consequence of head motion and the vestibulo-ocular reflex were classified as fixations [51]. After segmenting the fixations, the location of gaze was surrounded by a 60×60 pixel fixation window (approximately 2×2 degrees) such that for every frame, the location of gaze was projected on a 2D space. Using this method, we polled the virtual world and identified the objects that were fixated by the subject. Brief track losses during a fixation were ignored when the gaze location was on the same object before and after the track loss.

Data were then segmented into trials. A trial began when the subject was instructed to touch a particular object indicated by the name at the bottom of the screen (for example, Figure 1C), and ended as soon as the subject had “touched” the target. Since a substantial portion of the trial was dedicated to moving the subject's body from one room or location to another, each trial was subdivided into three segments: time from trial start until the object appeared in the subject's field of view, the subsequent period until the object was fixated, and the time between fixation and touching. We restricted our analyses (unless mentioned otherwise) to a particular epoch most relevant to the visual search itself: from the moment a target appeared within the field of view (FOV), to the moment the target was fixated upon, followed immediately by a gesture to touch it. Video records were examined frame by frame to determine the exact time of trial end, which was defined as the first frame where the subject's hand began moving towards the object to “touch” it.

*Path Length*. One method to assess how well people learn spatial layout of the environment was by quantifying the path they took to reach a search target and comparing it to the shortest possible path they could have taken. For each trial, 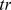, the length of the path 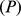 taken by the subject is measured using the following formula:
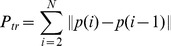
(1)where 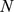 is the number of frames in that trial and 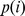 is the head position at frame 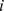. This value can then be compared to the shortest path for that trial 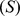,
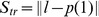
(2)where 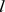 is the location of the target object. Notice that this measure does not account for walls and other obstacles but still provides a relative measure of performance on every trial.

To test the effect of repeated search on the number of fixations and path length, we performed a standard repeated measures ANOVA. Because the assumptions required for an ANOVA are often violated, however, we also performed a bootstrap version of the ANOVA which sidesteps most of the standard assumptions. For the bootstrap analysis we first computed the standard F-statistic for a repeated-measures design. Second, we generated the distribution of this F statistic under the null hypothesis where the repeated measures do not have an effect on the dependent variable. To generate this null-hypothesis distribution we resampled our data with replacement and ordered the data at random, thus eliminating the repeated-measure effect and calculated F in the standard way. We repeated this process 10000 times to generate 10000 F-statistics, yielding the F distribution under the null hypothesis. We then compared our F-statistic obtained in the experiment to the bootstrapped null distribution to compute the probability of obtaining such an F given the null hypothesis, which is akin to the standard measure of significance (comparable to the p-value usually reported in standard ANOVA analyses). In all of our analyses, the results of traditional ANOVA (corrected for sphericity using the Huynh-Feldt correction) agreed with those of the more conservative and assumption-free bootstrap analysis. We therefore report the ANOVA results throughout the text, as we feel it is more familiar to most readers.

## Results

### Distribution of fixations in the environment

**Figure 2:**
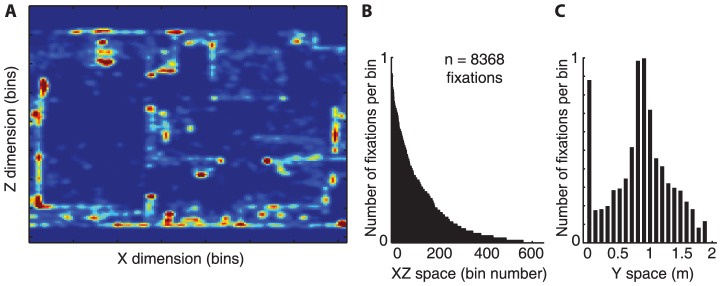
The distribution of gaze locations in the apartment on Day 1 reflect an inhomogeneous sampling of space. A. Heat map of gaze locations in the XZ plane, collapsed over vertical height, Y, within the apartment. B. Distribution of fixations in the XZ plane, ordered by frequency and normalized. C. Histogram of gaze locations on the Y axis, vertical height, normalized. Presented data are grouped over all six subjects for Day 1.

Since little is known about the characteristics of gaze deployment in natural immersive environments we first present summary data showing the regions fixated during exploration of the environment. Approximately 2,000 fixations per subject were recorded on each of the first two days. Figure 2A presents the distribution of subjects' gaze points within the virtual apartment in heat map form, on the first day. Gaze points for all subjects were included in this analysis to represent the average gaze behavior across subjects. After collapsing gaze points over height (Y), each point within the area of the apartment (XZ space) was replaced by a 2D Gaussian with a standard deviation of 5 cm. The Gaussians were then summed at each XZ location and the resulting image normalized. The heat map reveals the structure of the room and the location of the counters where many objects were located, and the edges of the doorways that are presumably fixated when moving between rooms. The wall structures that are picked out in the heat map may reflect gaze location “en passant” as the subject moved around the room. Figure 2B shows an ordered histogram of fixation frequency in the XZ space for all subjects. Figure 2C shows a histogram of the distribution of fixation heights (Y value) for all subjects and shows that gaze is largely concentrated on mid-height regions of the rooms, especially the horizontal surfaces. Additionally, there is an increased density of fixations on the floor, and in general the distribution is biased towards the lower part of the room. Note that the bulk of the distribution in both XZ space and height is contained within a small region of the 3D space. That is, many regions have either no fixations or very few fixations. Thus memory representations presumably will reflect this inhomogeneous sampling of space. A substantial component of this distribution can be attributed to the location of the search targets in the scene, but it presumably also reflects the subjects' priors about where everyday objects might be. Note that the distributions will reflect the general instruction to subjects to familiarize themselves with the environment in addition to the task of searching for specific objects. These distributions may not be entirely natural, of course, since the HMD restricted the vertical field of view, and subjects may limit their vertical head movements because of the weight of the helmet. The data are, however, consistent with the kinds of priors observed in inspection of 2D images of scenes [52]. In Figure 2 we present the distribution of gaze and fixations over all subjects, but we note here that qualitatively, the individual subject distributions were strikingly similar between subjects and of course to the mean across them.

### Visual Search

The main task in the experiment was for the subject to find objects in the environment. The search process first involves getting the body to the correct room, close to the object, and then locating it visually when it appears on screen. Rather than having a single measure of search, we broke it down into two components. The first was the number of fixations to locate the target once it came on within the field of view of the subject, and the second was a measure of how efficiently the subject moves the body into the correct location. Measuring the total number of fixations from trial start to trial end would be dominated by the relatively long time it takes to transport the body from one room to the next, and not address visual search per se. We therefore measured two aspects of search separately.

**Figure 3:**
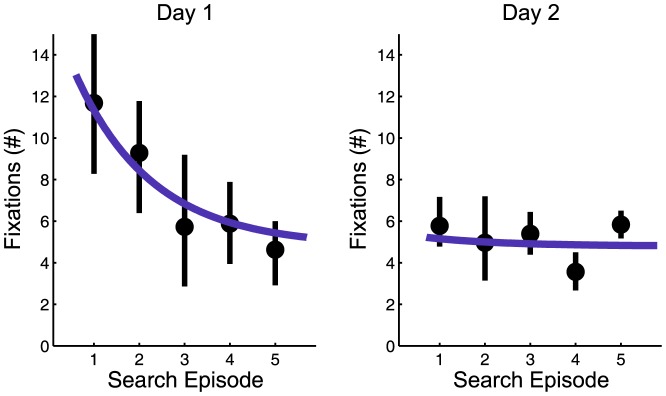
The number of fixations required to locate a search target decreases over repeated Search Episodes. Visual search performance is quantified as the number of fixations allocated in space between when the search target appeared in subject's field of view to the time the target was fixated upon. Data are for the three objects that were repeatedly searched for, averaged first over objects and then subjects, for day 1 and day 2. Error bars represent 95% confidence intervals, bootstrapped, between subjects. The curves show an exponential fit over the two days.

To quantify visual search performance over time we measured the number of fixations allocated by the subject to locate the object, once the image of the object was in the subject's field of view and visible. The number of fixations was counted between the time the target object came on screen and the time fixation landed on that object. We treat each object independently and consider the first time each of the 3 objects became the target of a search as Search Episode 1. The same was done for Search Episode 2, 3, 4, and 5. Figure 3 shows the average number of fixations as a function of Search Episode (first, second, third, etc.), averaged first over objects and then over subjects. The three objects that were used as a target repeatedly were selected for this analysis. The figure shows that by Search Episode 3 the number of fixations has dramatically decreased from approximately 12 fixations to 5–6. The plot for the second day shows little improvement over the first day. Thus, this measure of search shows very rapid learning of the object's location in the environment. A repeated-measures ANOVA showed a significant effect of Search Episode on Day 1, F(4,20) = 8.34, p = 0.002. In addition to Search Episode, a significant effect was also found for the search-object, F(2,10) = 7.101, p = 0.01. Thus, some objects required more fixations to locate them than others. This may be a consequence both of visual factors and also the fact that there was an unequal amount of experience in the environment for different objects. There was also a small Search Episode and search-object interaction effect, F(8,25) = 2.29, p = 0.053), suggesting that the magnitude of decrease in search fixations may be different for different objects, albeit only marginally significant. Since the trials for repeated objects were interspersed with searches for other objects, subjects had the opportunity to become familiar with the context, so contextual learning may contribute to the facilitation of search over the 5 episodes, in addition to learning the location of the object within the scene. As for Day 2, there were no significant effects for either Search Episode, search-object, or interaction (F(4,20) = 3.98, p>0.05; F(2,10) = 0.79, p>0.05; and F(8,19) = 0.24, p>0.05, respectively).

**Figure 4:**
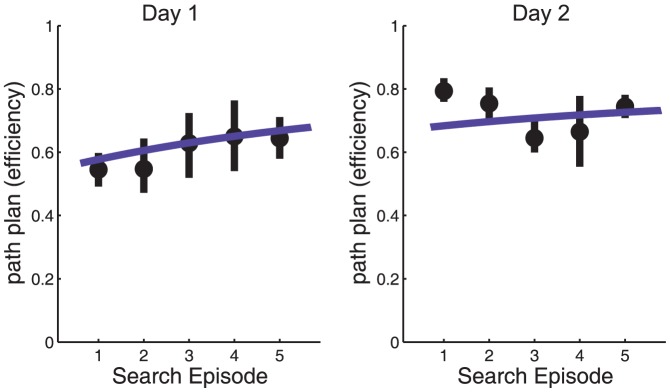
The efficiency with which subjects plan their path to search targets modestly increases over repeated Search Episodes. Path efficiency (the shortest possible path between subject and search target divided by the actual path taken by the subject to search target, see methods) is plotted as a function of Search Episode. The path is calculated between trial start to the time the target was fixated upon. Data are for the three objects that were repeatedly searched for, averaged over objects and subjects, for day 1 and day 2. Error bars represent 95% confidence intervals, bootstrapped, between subjects. The curves show an exponential fit over the two days.

To evaluate the more global aspects of search performance we developed a metric for path planning efficiency by calculating the ratio between the shortest path between subject and object at the beginning of a trial, and the path the subject actually traveled (detailed in the Methods). A value of 1 is the shortest possible path, and small values indicate a circuitous route (by definition, an efficiency of 1 is never possible as it ignores walls.) Path efficiency is plotted in Figure 4 as a function of Search Episode. Similarly to Figure 3, path efficiency was first averaged over the three objects that were repeatedly searched for, followed by averaging over the subjects. Figure 4 demonstrates a fairly gradual increase in path planning efficiency over Search Episodes. By the end of the second day the mean path planning efficiency increased to about 0.75, equivalent to a 35% decrease in path length to the search target. However, the apparent increase was not statistically significant in a repeated-measures ANOVA for either Search Episode, search object or interaction for Day 1 (F(4,20) = 1.85, p>0.05; F(2,10) = 1.98, p>0.05; and F(8,33) = 0.4, p>0.05, respectively) or for Day 2 (F(4,20) = 2.69, p>0.05; F(2,10) = 0.47, p>0.05; and F(8,30) = 2.35, p>0.05, respectively). Note that even for the first Search Episode, the subject had been in the environment for several minutes searching for other targets, and so had multiple opportunities to learn the general arrangement of the apartment within that period and presumably could select the correct room on the basis of semantic information.

### Effect of task irrelevant fixations on subsequent search

**Figure 5:**
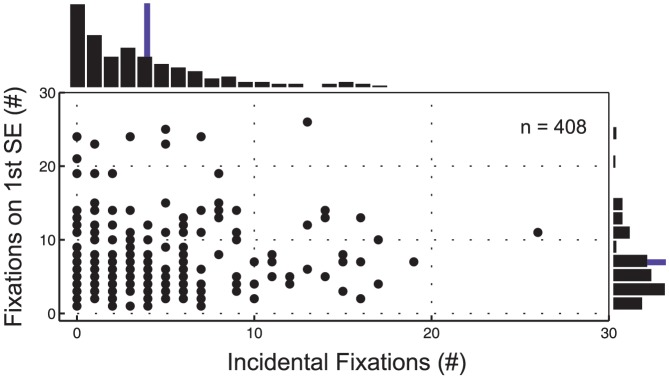
The number of incidental fixations to objects that have yet to be searched for does not correlate with number of fixations required to locate the object on 1st Search Episode. Incidental fixations (x-axis) are considered incidental if the fixation was made to a non-target object before that object has ever been identified as a search target. The number of fixations required to locate the object once it has become a search target for the first time (1st Search Episode) are presented on the y-axis. Each object contributes 1 data point, and identical points are not obvious on the scatterplot. Marginal histograms are therefore presented to the right and above the scatterplot, and distribution means are indicated by the thin lines. SE =  Search Episode.

Subjects clearly learn the location of objects in the environment very rapidly. However, is this a consequence of the fact that they have been explicitly attended, or incidentally attended to? In the course of a session, subjects make thousands of fixations. Do incidental fixations also lead to an accumulation of memory representation? To examine this question we measured the number of incidental fixations on objects that had not yet been explicitly searched for. We then measured the number of fixations allocated by subjects to locate the object on the first occasion that it became a search target (i.e. Search Episode 1). If subjects accumulate memory from the incidental fixations we would expect to see more rapid or facilitated search as a consequence. Figure 5 presents the relationship between incidental fixations to objects before they have ever been considered search items and the number of fixations required to locate the same object on the 1st Search Episode. For this analysis the entire dataset was used for all objects. There does not appear to be any trend in the data for increased numbers of incidental fixations to lead to more rapid search. A regression line fitted to the data (not shown) has a slope close to zero, and the correlation coefficient of r = 0.09 was not significant (p = 0.175). Although there was no trend in the regression, it is hard to make the case that search does not benefit either from incidental fixations or from experience in the environment. On the whole, performance is very variable. Thus many objects were found rapidly, with 5 or fewer fixations, even if they had not fixated the object previously, whereas others required 20 or more fixations to be located, despite having ten or more previous incidental fixations. It appears that some targets may be remembered from prior fixations whereas others do not reveal a search benefit. Thus the number of incidental fixations alone does not seem to be a primary causal factor in memory in this task.

### Change Detection

**Figure 6:**
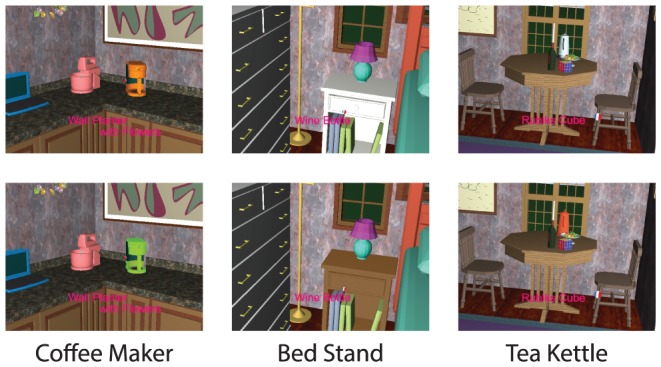
The three objects that were searched for repeatedly and their color change on day 3 of the experiment. From left to right: the coffeemaker, bedstand, and kettle. Top row presents the object as it was on day 1, day 2 and day 3 before change, bottom row presents the objects after the day 3 change.

**Figure 7:**
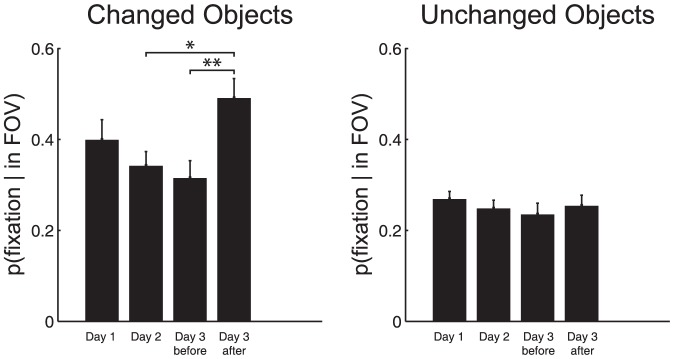
The probability of fixating an object increases for objects that have changed color, but not for those that have remained unchanged. Both panels present p(fixation|inFOV), the probability of fixating an object given that it is in the field of view and not a current target of a search, for Day 1, Day 2, Day 3 before the color change and Day 3 after the color change, averaged over objects and subjects. A. Mean p(fixation|inFOV) for the three objects that changed color. B. Mean p(fixation|inFOV) for the remaining unchanged objects. Error bars are standard error between subjects. FOV =  field of view. * p>0.05, ** p>0.001, corrected for multiple comparisons.

One of the main goals of the experiment was to test whether experience in the apartment increased the probability that subjects would fixate the changed region. On the third day of the experiment, subjects continued to search for objects, but a change was introduced. The three objects that were chosen for repeated searches (coffee maker, kettle, bed stand) were each searched for once, and then changed color (at different times, see Methods for details). The changes are shown in Figure 6. Day 3 was terminated on the 60th trial, and subjects then filled out the questionnaire. To quantify whether the change drew attention we calculated the probability of fixating each of the three objects during the periods when that object was in the subject's field of view, but was not the target of a search. This probability was calculated both before and after the change. A value of 0 means that even though the object was on screen it was never fixated, while a value of 1 implies that it was fixated at least once during each episode when the object was on screen. Figure 7A summarizes these fixation probabilities, together with the fixation probability for the first two sessions (day 1 and 2). Over the first three sessions, a steady (but non-significant) decrease in fixation probability is observed. Once the change was introduced, there was an increase in the probability of fixating the changed object, from 0.31 to 0.49. A one-way repeated-measures ANOVA showed there to be a significant effect of search epoch (day 1, day 2, day 3 before and day 3 after change) on the probability of fixating on an object given that the object has entered the field of view,F(3,51) = 9.29, p<0.001, corrected. A posthoc repeated-measures ANOVA revealed significant differences between the probability of fixation on day 2 compared to day 3 after (p<0.05) and between day 3 before and day 3 after (p<0.001), corrected for multiple comparisons. Figure 7B shows the same computation for 17 control objects that were not changed, and were comparable to the three repeated objects in size, location, and in probability of entering the field of view. A similar modest decrease in of fixation probability is observed between day 1 and day 3, but in contrast to the objects that changed color, there is no substantial increase in probability after the change. A one-way repeated ANOVA found no significant effect of time on the probability of fixating an object given that the objects has entered the field of view,F(3,303) = 1.43, p>0.05. A similar non-significant result was found regardless to whether this analysis included the 17 “comparable” objects or the full array of objects.

**Figure 8:**
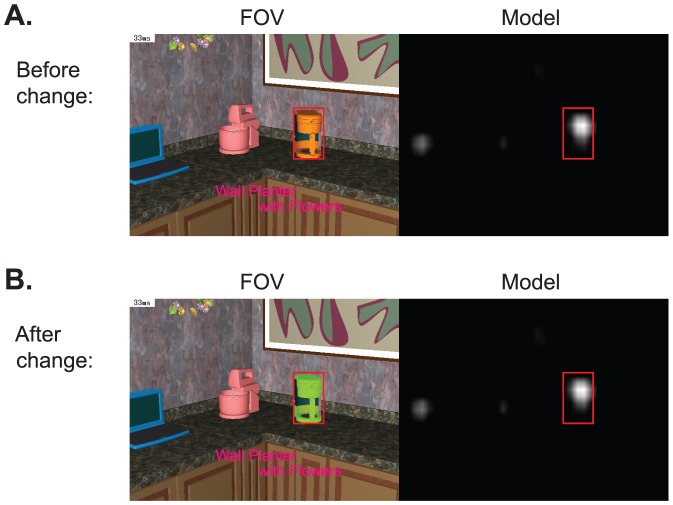
Bottom up saliency does not change as a result of object color change. Left: Image of the coffee maker and corresponding saliency map per code by Itti and Koch [22]. Right: Image of the coffee maker after the color change, and the corresponding saliency map. Saliency values were computed within the red rectangle.

It is possible, of course, that the color changes that were introduced increased the bottom up salience of the targets. To evaluate this, we used the code provided by Itti et al. [22] at http://ilab.usc.edu/toolkit/downloads.shtml to calculate the salience maps before and after the color change. A virtual camera was placed at a location where the desired object was fully in view. The world was rendered twice. Once with the original appearance of the object and once with the color changed. The *ezvision* executable with default parameters was executed separately against both scenarios. A bounding box was placed over the object in the saliency map and the pixel values (ranging from 0 to 255) were summed up to calculate the final saliency score. The scene and the corresponding saliency maps for an example object, the coffee maker, are shown in Figure 8. The saliency value for the kettle and the bed stand decreased after the color change, and saliency for the coffee maker increased by only 3%. Thus the increase in fixation probabilities are unlikely to be the result of an increase in bottom up salience.

## Discussion

The goal of this experiment was to study the role of scene memory in visual search and change detection in an immersive environment. The experiment required subjects to spend a prolonged period of time in the environment in order to provide an exposure more comparable to natural experience than standard experimental paradigms. Time in the environment was necessarily limited, and amounted to a little more than an hour over three days. However, within that period subjects each made over 10,000 fixations within the virtual apartment. Consequently the visual experience parallels at least a subset of ordinary experience. We found that subjects in such environments confine their gaze almost exclusively to mid-heights, with almost no fixations on high regions in the environment. Part of the predominance of mid-height fixations is explained by the location of the search targets, but the absence of high or low fixations (except for the floor) indicates that subjects typically do not explore such regions, and suggests the existence of strong priors for where the search targets are likely to be located. This is consistent with the finding of such priors in 2D natural scene images by Torralba et al. [52]. We were not able to discern any obvious changes in the spread of fixations within the environment with experience.

In an attempt to separate the global and local aspects of search we looked at two components of the search epoch separately. The global component was assessed by measuring the efficiently by which subjects approached the search target over the course of a trial. We found that path efficiency had measured the length of the path taken until the object appeared on screen, relative to the shortest direct distance from the start of the trial to the object. Path efficiency gradually improved only a modest amount over repeated searches during the first session. Thus, finding the approximate global location did not change very much over repeated searches. However, the subject had been in the environment for several minutes searching for other targets, and so had multiple opportunities to learn the general arrangement of the apartment (kitchen and dining area, bedroom, bathroom), and may have moved to the approximate location on the basis of semantic cues, such as moving to the kitchen for the coffee maker. Once in the correct room the subject need only orient the head in the correct direction in order to bring the target on screen.

The local component of search was assessed by measuring the number of fixations made by the subject from the moment the search target had entered subject's field of view and until successful location of the target. This local aspect of search improved rapidly with repeated search, falling from about 12 to 5 fixations and stabilizing after 3–5 episodes, with most of the improvement occurring between the first and third Search Episodes. This suggests that memory for spatial location is an important factor in locating targets in natural circumstances. It is also possible that memory for visual features linked with the verbal description facilitated search. This memory persisted when subjects repeated search on the next day, with little if any detectable memory loss. It is hard to make precise comparisons with other work in the literature, given the very different experimental context. However, the finding of rapid improvement in performance with repeated search is consistent with the findings of Võ et al. [53], Hollingworth [50] and others, although the number of fixations to locate the object once on-screen is somewhat greater in our task (five fixations versus 1 or 2). Once the target is on screen, the primary difference in the conditions is that in the immersive case the scene varies with head and body movements, whereas there is a single fixed image in the standard 2D case. Additionally, the subject may need to devote some attentional resources to locomoting in the environment [54].

In the context of the repeated searches, we assessed memory for items that had been explicitly attended. However, to understand the development of scene memory we need, in addition, to know whether subjects encode the locations that they fixate in the context of other searches as well, when they are not explicitly relevant. Do incidental fixations contribute to future searches for other objects? To investigate this we looked at the number of fixations required to locate an object when it first became a search target, as a function of the number of prior incidental fixations on the object during the preceding period. We found no relation between first search time and number of incidental fixations. Thus incidental fixations are neither necessary nor sufficient. This result is similar to that observed by Võ et al. [53] and may reflect a variety of factors. First, the presentation of search target in verbal form doubtless works against an effect of prior fixations, as subjects may not connect the visual and verbal representations [49]. Second, there is a lot of intrinsic variability. Some targets are found easily with few or no prior fixations, and others are difficult to locate even with 10–20 prior fixations. It seems clear that number of prior fixations is not the most important variable. It may be the case that some items are fixated and remembered, and others forgotten. Whether an item is remembered or not may depend on the subjects' knowledge of whether the item needs to be remembered or is task relevant. This possibility is supported by observations made by Tatler et al. [44] in a real world setting: subjects instructed to look for items related to tea making remembered those items, but with only a general instruction to remember the objects, subjects remembered those items less well, even when they had fixated them an equal number of times. Thus a critical factor might be some knowledge of the probability that the information will be needed in the future, rather than the fixation event itself. Another important factor is the existence of strong semantic guidance which may make the search easy for some objects [49], such that memory-based search is not the limiting factor. Thus although scene previews undoubtedly lead to the development of memory representations (e.g., [47], [50], [55]), semantic context effects may be of greater benefit in many circumstances, as are explicit task relevant fixations.

A final goal of this investigation was to study whether the development of memory representations might form the basis of an exogenous attention mechanism. While endogenous mechanisms can account for much gaze behavior, any account of gaze control will be incomplete without some mechanism to attract gaze to unexpected stimuli. We therefore made color changes in a small number of objects after extensive experience in the environment, and measured whether the unexpected change had increased the probability of attracting gaze. We found a significant increase in fixation probability that was not observed in control objects. Given the kinds of prominent changes in scenes that frequently go undetected (e.g., [4]), we might expect that changing the color of a single object in a complex immersive scene would be a very weak stimulus for attracting gaze, so this result provides some evidence for the hypothesis that more extensive memory representations enhance the detectability of changes. As discussed in the introduction, other lines of evidence also indicates that more elaborate memory representations increase the probability that subjects will fixate changed regions [39]–[42]. The present results extend those findings to the kinds of situation that the visual system typically needs to cope with, where experience is built up over extended periods in the same environment. They also extend the results of Karacan et al. [46] by showing an influence on fixation probability, not just fixation duration. The data provided in this experiment are necessarily sparse, since we are looking for a single event: does a change in the environment evoke a fixation in an uncontrolled situation where many factors might be controlling the subjects' attention. This is the kind of situation the visual system must deal with. There is typically only a limited time window when gaze needs to be attracted to some event of importance such as an unexpected step or a crack in the pavement, so the question is intrinsically difficult to resolve. In addition, only three objects were changed, there were only six subjects, and the increment in fixation probability was only about 0.2. Therefore the result is not a very strong one, but on the whole supports the suggestion that memory based expectations may be a factor in detecting environmental irregularities. This extends Itti's et al. idea of Surprise to contexts where the comparison base is a long-term memory representation.

One of the motivations for investigating change detection in immersive environments is to study the nature of memory representations that develop during natural experience. Detecting changes in video streams has been an active area of study in computer vision in the context of surveillance (e.g. [56]–[58]) but most of these efforts have been on stationary cameras, where the solutions are relatively straightforward. For mobile cameras that have no constraints in the way they move through the environment, these techniques do not work. This is more like the situation humans face when moving through an environment. Without a more complex memory model, changes that happen between visits cannot be detected by these models. The Itti and Baldi [33] model discussed in the introduction is one such attempt. Another attempt makes use of the color signature of the scene, which is tolerant to moderate viewpoint changes [59]. In this model RGB color histograms provide scene specific signatures that are largely view and resolution independent [60] at least in indoor environments that are potentially suitable for change detection in natural vision. The Kit et al. [59] model is trained on histograms of image sequences generated by subjects as they explore environments. This data is then mapped onto a much smaller number of memory units using an unsupervised clustering algorithm. This model was able to detect the changes in colors of the objects in the scenes used in the current experiment [61]. It is clear that human scene representations are much more complex than color histograms, so this is not necessarily an indication of the representations humans use. However, the success of this simple model in detecting changes in dynamically varying views demonstrates that humans may be able to develop robust change detection mechanisms from quite simple memory representations.

## Conclusion

In summary, we found that in a naturalistic immersive environment, scene memory plays an important role in visual search and may serve to facilitate change detection. Subjects distributed gaze over a restricted portion of the 3D space, perhaps reflecting priors from previous experience. In agreement with previous evidence form standard 2D paradigms, subjects quickly learn the location of objects in space. Both global and local measures of search suggest that experience in the environment better guides search, and improvements are observed already after one or two Search Episodes. When search targets are specified by verbal labels, incidental fixations do not appear to be a primary determinant in facilitating subsequent search. Other factors such as semantic information about the environment may guide search more efficiently as suggested by Võ et al. [49] or memory may decay rapidly when the need for the information is not clearly specified. We also found reliable evidence that after 3 days of experience, modest changes in the scene such as changing the color of an object was able to attract gaze, supporting previous evidence for memory-guided prioritization. Thus an important function for visual memory is to serve as a basis for a robust Surprise mechanism, and to increase the probability that novel or unusual features of a scene will attract gaze. Such a mechanism is a necessary adjunct to both task-guided gaze allocation and simple feature-based saliency.
